# Some Modern Continental Hospitals

**Published:** 1908-09-26

**Authors:** 


### HOSPITAL ADMINISTRATION.

### CONSTRUCTION AND ECONOMICS.

### SOME MODERN CONTINENTAL HOSPITALS.

THE GYNÆCOLOGICAL CLINIC AT GREIFSWALD.—II.

Not the least excellent feature of the reconstructed and modified "Frauenklinik" at Greifswald is its fine educational side. In the first article we briefly described the accommodation for patients and what may be styled the purely hospital part of the institution. It remains still to describe the other—its educational side—and to show in how far the clinic can be considered a modern and up-to-date teaching establishment.

When the reconstruction of the old clinic was taken in hand it was decided that the new building should not only be a model gynæcological and obstetrical hospital, but that it should at the same time serve as a model teaching and demonstration clinic. The amount of material in this department at the disposal of the Greifswald faculty is not, indeed, to be compared with what is to be found at large centres, but there is sufficient material at hand to make it possible for the courses in midwifery and diseases of women to be as good and as thorough as they are at most smaller German Universities, and certainly, in many respects, far superior to what obtains at our London medical schools, where the gynæcologist has only one ward, and not many cases for demonstration purposes. The Greifswald clinic has not only to serve a much larger number of students than that attending any London school, but it has also to train and educate a number of midwives. This has made it necessary to establish a school of midwifery for nurses along with the usual medical midwifery school; but here, as in other respects, the directorate has enforced a rigid separation between the classes. The students have a special entrance and special rooms, while the nurses and probationers, who are provided with accommodation in the building, have their own special staircase, and the lectures are given at different periods. Those students who are "Haus-praktikauten," or internes, have their room in the main building. They have a strict rota of service, and take turns at "observation," "attendance," and "reporting." The observation room destined for their use is on the first floor, close to the delivery room, and is comfortably furnished with sofa, couch, reading table, and easy chairs—a veritable palace when compared with the dens in which some of our London "externs" still have to exist while on "midder duty." The German extern usually lives close to the hospital, and does district duty for several months, although not so continuously nor so strenuously as the London man. Equally comfortable are the rooms for the resident medical staff. The officer in charge of each division has his rooms quite close to the division of which he is in charge, and equally close to the sisters' room. The director's study adjoins the main lecture theatre, and immediately behind it is the physician's common room, or Camis, as it is called, where a small reference library and the museum are kept. Demonstrations are given in the theatres, both in the septic and in the ordinary operating theatres, and in the delivery rooms. In addition there is a large theoretical demonstration room. On the first floor the greater part of the west wing is ocupied by the laboratory, which consists really of three large rooms. The first of these is the large and airy examination room, while the other two form what is called the demonstration gallery. These rooms are adequately provided with light and water, and are well suited for their purpose. The main demonstration room is, however, the large and fine clinical lecture theatre, which accommodates 110 students. Although this room—with its fine open windows admitting so much light, and its terrazzo floor, tiled walls, and iron fittings enamelled white—is very suitable for operative work, it is rarely used for that purpose, the demonstrations being usually purely clinical. Patients are brought in on "Ruhebetten," or bed-couches, which are wheeled into the theatre. Such patients are then demonstrated to the class, or students are called down to make the usual examinations in a more thorough and, therefore, much more satisfactory manner than would be possible in ward work. The theatre is provided with the usual apparatus for demonstration purposes—screens for epidiascopic pictures, instruments, and all necessary addenda. From this theatre one enters the preparation rooms, which include an anæsthetising and a dressing room. By means of the corridor they are in connection with the wards, thus facilitating the removal of patients. The electric lift is also close at hand and is another convenience.

The clinic has an out-patient department for gynæcological patients which is well attended. The out-patient entrance is situate at the east end of the building, and quite separate, therefore, from the main and the students' entrances. Adjoining this entrance is a small lobby which leads into a commodious waiting-room, attached to which is the usual lavatory accommodation. The main out-patient room is large and airy, provided with three large windows, and fully equipped. Both gas and electric light are used, and the arrangements for instruments, cards, and letters is excellent, so that little time is wasted. Attached is a preparation room. The examination couches are arranged at the end of the main room, which is provided with a series of double screens to assure privacy. In the demonstration theatre the room can be completely darkened by means of an automatically acting screen, as is the usual arrangement in most modern lecture theatres.

There is a small building at the extreme north-western end of the building devoted to the animals used for experimental purposes. This building is excellently arranged, the isolation stalls and observation rooms being quite distinct from the ordinary kennels. In England, where comparatively little experimental work is done that touches upon vivisection, such an institution is nowhere to be found. In all Continental hospitals, however, the animal stable is an existing reality which has to be duly considered by the architect in designing the institution.

The admission of patients is either directly through the main entrance, or indirectly through the out-patient department. In the former case the patients are usually either paying patients, who go to one of the class-rooms (these must not be confounded with the lecture theatre), or "from the district" where they have already been under observation. In either event their admission is a matter of comparative urgency. In the second case the patients who are to be admitted from out-patients are taken in hand by the nurse on duty, and are brought either to the gynæcological or to the obstetrical department, as may be necessary. The midwives who are being trained in the institution number twenty-four, and have their quarters above the auditorium. There they have two dormitories, and one large day or recreation room. About a third of this number are usually engaged in the wards during the day. Lectures and demonstrations are regularly given, both by the medical staff on the usual subjects, and by the matron on gynæcological and obstetrical nursing.

The Frauenklinik at Greifswald, as may be seen from this short description, is a thoroughly practical and modern institution. Were the number of patients admitted more, or the material with which it has to deal greater, the institution might be criticised as being too small. As it is, there is nowhere any overcrowding. As has been stated, the wards are roomy, and the amount of bedspace provided is far in excess of the average. The institution is admirably managed, and may be looked upon as a model of its kind. As it is one of the newest of gynæcological clinics it will, of course, have to bear comparison with other similar modern gynæcological clinics, and if we take, for purposes of comparison, the only just completed and not yet occupied gynæcological department of the large general hospital at Vienna, we may state that such comparison is in no way detrimental to the smaller German clinic.

